# Emodin targets the β-hydroxyacyl-acyl carrier protein dehydratase from *Helicobacter pylori*: enzymatic inhibition assay with crystal structural and thermodynamic characterization

**DOI:** 10.1186/1471-2180-9-91

**Published:** 2009-05-12

**Authors:** Jing Chen, Liang Zhang, Yu Zhang, Haitao Zhang, Jiamu Du, Jianping Ding, Yuewei Guo, Hualiang Jiang, Xu Shen

**Affiliations:** 1 Drug Discovery and Design Center, State Key Laboratory of Drug Research, Shanghai Institute of Materia Medica, Chinese Academy of Sciences, Shanghai 201203, PR China; 2 Institute of Biochemistry and Cell Biology, Chinese Academy of Sciences, Shanghai 200031, PR China

## Abstract

**Background:**

The natural product Emodin demonstrates a wide range of pharmacological properties including anticancer, anti-inflammatory, antiproliferation, vasorelaxant and anti-*H. pylori *activities. Although its *H. pylori *inhibition was discovered, no acting target information against Emodin has been revealed to date.

**Results:**

Here we reported that Emodin functioned as a competitive inhibitor against the recombinant β-hydroxyacyl-ACP dehydratase from *Helicobacter pylori *(HpFabZ), and strongly inhibited the growth of *H. pylori *strains SS1 and ATCC 43504. Surface plasmon resonance (SPR) and isothermal titration calorimetry (ITC) based assays have suggested the kinetic and thermodynamic features of Emodin/HpFabZ interaction. Additionally, to inspect the binding characters of Emodin against HpFabZ at atomic level, the crystal structure of HpFabZ-Emodin complex was also examined. The results showed that Emodin inhibition against HpFabZ could be implemented either through its occupying the entrance of the tunnel or embedding into the tunnel to prevent the substrate from accessing the active site.

**Conclusion:**

Our work is expected to provide useful information for illumination of Emodin inhibition mechanism against HpFabZ, while Emodin itself could be used as a potential lead compound for further anti-bacterial drug discovery.

## Background

*Helicobacter pylori *(Hp) is one kind of rod- or curve-shaped and microaerophilic gram-negative bacterium that is located along the surface of the mucosal epithelium or in the mucous layers [1]. It has been recognized as a major causative factor for several gastrointestinal illnesses of human, such as gastritis, peptic ulceration, and gastric cancer [2]. *H. pylori *has become a severe threat against human health, and probably chronically infected about 50% of the world's human population [3]. Currently, the combination therapy is still regarded as the most effective treatment against *H. pylori *infection [4]. However, the overuse and misuse of antibacterial agents have resulted in the alarming rise of antibiotic-resistant strains [5]. Thus, novel antibacterial agents acting on new targets are needed urgently. Fortunately, due to the major difference between the enzymes involved in the type II fatty acid synthetic pathway (FAS II) in bacteria and the counterparts in mammals and yeast, the enzymes involved in FAS II has been treated as potential antibacterial drug targets [6]. Of the important enzymes for the elongation cycles of both saturated and unsaturated fatty acids biosyntheses in FAS II, β-hydroxyacyl-ACP (FabZ) has attracted close attention as an essential target for the discovery of effective anti-bacterial compounds against pathogenic microbes [6]. Recently, FabZ from *H. pylori *strain SS1 (HpFabZ) was cloned and purified [7]. The further HpFabZ enzymatic characterization and the crystal structures of HpFabZ and its complexes with two inhibitors [7,8] have provided valuable information for HpFabZ targeted anti-*H. pylori *agent discovery.

**Figure 1:**
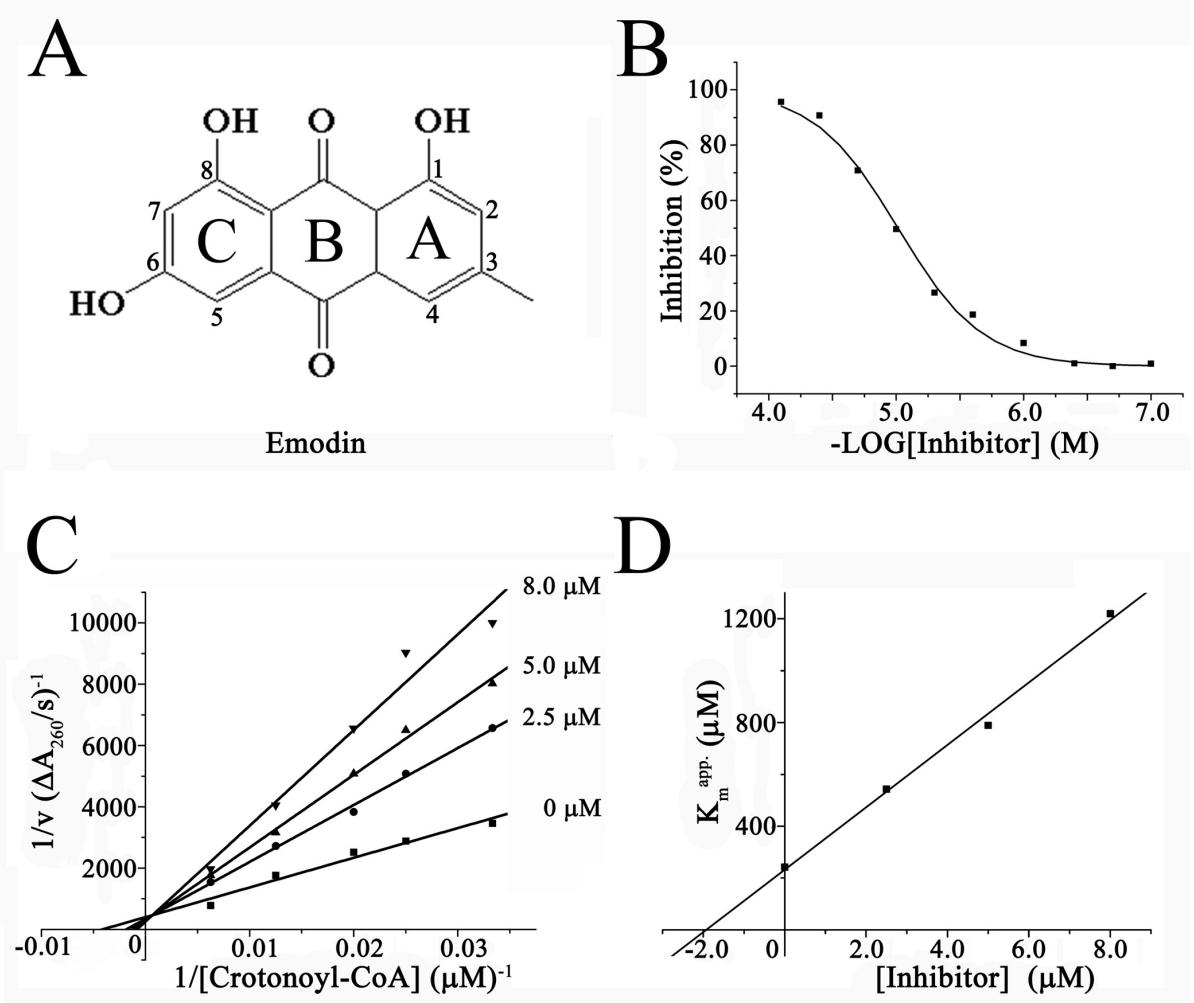
**(A) Chemical structure of Emodin**. The three rings are named and their positions are numbered according to the nomenclature. (B) Dose-response curves for enzyme inhibition (IC_50 _= 9.70 ± 1.0 μM). (C) Kinetic analysis of Emodin inhibition against HpFabZ. The panel shows the representative double reciprocal plots of 1/V vs 1/[Substrate] at different inhibitor concentrations. The lines intercept on the 1/V axis, indicating that Emodin is a competitive inhibitor for the substrate crotonoyl-CoA. (D) Secondary plot of *K*_m_. The inhibition constant *K*_i _is 1.9 ± 0.3 μM.

The natural product Emodin (3-methyl-1, 6, 8-trihydroxyanthraquinone, Fig. 1A) is originally isolated from the rhizomes of Rheum palmatum. It exists in the roots and bark of numerous different traditional Chinese medicine (TCM) formulations and Chinese medical herbs such as Rheum officinale Baill (Polygonaceae), Rhamnus (Rhamnaceae), and Senna (Cassieae) [9]. Emodin demonstrates a wide range of pharmacological properties such as anticancer [10], anti-inflammatory [11], antiproliferation [12], and vasorelaxant activities [13]. It has been reported that Emodin has a regulatory effect on the proliferation of human primary T lymphocyte [14] and immune responses in human mesangial cells [15], inhibits the proliferation of pancreatic cancer cell through apoptosis induction-related mechanism, accelerates osteoblast differentiation through phosphatidylinositol 3-kinase activation and bone morphogenetic protein-2 gene expression [16]. It could also inhibit the growth of neuroectodermal cancer [17] and breast cancer by suppressing HER-2/neu tyrosine kinase activity in HER-2/neu-overexpressing human breast and lung cancer cells [18-20], inhibit tyrosine-kinase-mediated phosphorylation of vascular endothelial growth factor (VEGF) receptors in colon cancer cells [21], promote the repair of nucleiotide excision to the DNA damage of human cells caused by UV and cislatin induction [22], and finally competitively block the activity of casein kinase II [23]. In addition, Emodin was previously reported to show inhibitory activity against the growth of *Helicobacter pylori *by inducing dose-dependent DNA damage [10]. However, no acting target information for Emodin inhibition against *H. pylori *has been revealed to date.

In the present work, we reported that Emodin functioned as a competitive inhibitor against HpFabZ. In order to further study the inhibitory mechanism, the kinetic and thermodynamic characterization of Emodin/HpFabZ interaction was investigated by surface plasmon resonance (SPR) and isothermal titration calorimetry (ITC) based assays. In addition, the crystal structure of HpFabZ-Emodin complex was also determined to inspect Emodin/HpFabZ binding at atomic level. Our work is expected to have provided useful information for illumination of the possible Emodin inhibition mechanism against HpFabZ, while Emodin could be discovered as a potential drug lead compound for further research.

## Methods

### Materials

Standard *H. pylori *strains SS1 and ATCC 43504 were obtained from Shanghai Institute of Digestive Disease. *E. coli *strain BL21 (DE3) was purchased from Stratagene. All chemicals were of reagent grade or ultra-pure quality, and commercially available.

### HpFabZ enzymatic inhibition assay

The expression, purification and enzymatic inhibition assay of HpFabZ enzyme were performed according to the previously published approach [7,8] with slight modification. The compounds dissolved in 1% DMSO (Dimethyl sulfoxide) were incubated with the enzyme for 2 hours before the assay started. The IC_50 _value of Emodin was estimated by fitting the inhibition data to a dose-dependent curve using a logistic derivative equation. The inhibition type of Emodin against HpFabZ was determined in the presence of varied inhibitor concentrations. After 2h-incubation, the reaction was started by the addition of crotonoyl-CoA. The *K*_i _value was obtained from Lineweaver-Burk double-reciprocal plots and subsequent secondary plots.

### Surface Plasmon Resonance (SPR) technology based binding assay

The binding of Emodin to HpFabZ was analyzed by SPR technology based Biacore 3000 instrument (Biacore AB, Uppsala, Sweden). All the experiments were carried out using HBS-EP (10 mM HEPES pH 7.4, 150 mM NaCl, 3.4 mM EDTA and 0.005% surfactant P20) as running buffer with a constant flow rate of 30 μL/min at 25°C. HpFabZ protein, which was diluted in 10 mM sodium acetate buffer (pH 4.13) to a final concentration of 1.3 μM, was covalently immobilized on the hydrophilic carboxymethylated dextran matrix of the CM5 sensor chip (BIAcore) using standard primary amine coupling procedure. Emodin was dissolved in the running buffer with different concentrations ranging from 0.625 to 20 μM. All data were analyzed by BIAevaluation software, and the sensorgrams were processed by automatic correction for nonspecific bulk refractive index effects. The kinetic analyses of the Emodin/HpFabZ binding were performed based on the 1:1 Langmuir binding fit model according to the procedures described in the software manual.

### Isothermal titration calorimetry (ITC) technology based assay

ITC experiments were performed on a VP-ITC Microcalorimeter (Microcal, Northampton, MA, USA) at 25°C. HpFabZ was dialysed extensively against 20 mM Tris (pH 8.0), 500 mM NaCl and 1 mM EDTA at 4°C. Appropriate concentration of Emodin was prepared from a 50 mM stock in DMSO, and corresponding amount of DMSO (25%) was added to the protein solution to match the buffer composition. The reference power was set to 15 μCal/sec and the cell contents were stirred continuously at 300 rpm throughout the titrations. After an initial injection of Emodin (3 μL, not used for data fitting), 29 injections (6 μL each) were performed with a 3 min-delay between each injection, and then the heat changes were monitored. Blank titrations of Emodin into buffer were also performed to correct for the heats generated by dilution and mixing. The binding isotherm was fit by the single binding site model using a non-linear least squares method based on Origin (Microcal Software, Northampton, MA, USA).

### HpFabZ-Emodin complex crystallization and data collection

HpFabZ crystallization was performed using hanging-drop vapor-diffusion method similar to our reported approach [8]. 1 μl of HpFabZ (~10 mg/ml) in crystallization buffer (20 mM Tris-HCl, pH 8.0, 500 mM NaCl) was mixed with an equal volume of reservoir solution containing 2 M sodium formate, 0.1 M sodium acetate trihydrate at pH 3.6–5.6 and 2% w/v benzamidine-HCl. The mixture was equilibrated against 500 μl of the reservoir solution at 277K. When the dimensions of HpFabZ crystals grew up to 0.5 × 0.3 × 0.3 mm^3 ^after 7 days, Emodin was added into the original drops to a final concentration of ~10 mM and soaked for 24 hours. The crystal was then picked up with a nylon loop and flash-cooled in liquid nitrogen. Data collection was performed at 100K using the original reservoir solution as cryoprotectant on an in-house R-Axis IV++ image-plate detector equipped with a Rigaku rotating-anode generator operated at 100 kV and 100 mA (λ = 1.5418 Å). Diffraction images were recorded by a Rigaku R-AXIS IV++ imaging-plate detector with an oscillation step of 1°. The data sets were integrated with MOSFLM [24] and scaled with programs of the CCP4 suite [25]. Analysis of the diffraction data indicated that the crystal belongs to space group P2_1_2_1_2_1_.

### Structure determination and refinement

HpFabZ-Emodin complex structure was solved by molecular replacement (MR) with the programs in CCP4 using the coordinate of native HpFabZ (PDB code is 2GLL) as the search model. Structure refinement was carried out using CNS standard protocols (energy minimization, water picking and *B*-factor refinement) [26]. Electron density interpretation and model building were performed by using the computer graphics program Coot [27]. The stereochemical quality of the structure models during the course of refinement and model building was evaluated with the program PROCHECK [28]. The coordinates and structure factor of the HpFabZ-Emodin complex structure have been deposited in the RCSB Protein Data Bank (PDB code is 3ED0).

### Anti-*H. pylori *activity assay

The bacterial growth inhibition activity for Emodin was evaluated by using Paper Discus Method. DMSO and ampicillin paper were used as negative and positive control respectively. The minimum inhibitory concentrations (MIC) values were determined by the standard agar dilution method using Columbia agar supplemented with 10% sheep blood containing two-fold serial dilutions of Emodin. The plates were inoculated with a bacterial suspension (10^8 ^cfu/ml) in Brain Heart Infusion broth with a multipoint inoculator. Compound-free Columbia agar media were used as controls. Inoculated plates were incubated at 37°C under microaerobic conditions (85% N_2_, 10% CO_2 _and 5% O_2_) and examined after 3 days. The MIC value was defined as the lowest concentration of Emodin that completely inhibited visible bacterial growth.

## Results

### Inhibition of Emodin against HpFabZ

The recombinant HpFabZ enzyme was prepared according to our previously published report [7]. The spectrophotomeric enzyme inhibition assay approach [7,8,29] was used for randomly screening HpFabZ inhibitor against our lab in-house natural product library. In addition, to optimize the screening efficiency and creditability, the pH profile of HpFabZ and the potential effects of DMSO on enzymatic activity were investigated [see Additional files 1, 2 and 3]. As shown in Additional file 2: Fig. S1, the pH optimum of HpFabZ was 8.0 and 1% DMSO for dissolving the tested compound had no obvious effect on the enzymatic activity (Additional file 3: Fig. S2.)

Emodin was discovered as the inhibitor of HpFabZ by IC_50 _value of 9.7 ± 1.0 μM (Fig. 1B and Table 1) and further inhibition mode characterization suggested that it functioned as a competitive HpFabZ inhibitor with *K*_i _value of 1.9 ± 0.3 μM (Figs. 1C, D and Table 1). Similar to the other reported HpFabZ inhibitors [8,30], Emodin inhibited the enzyme activity by competing with the substrate crotonoyl-CoA.

**Table 1:** Inhibition summary of Emodin against HpFabZ and *H. pylori *strains

HpFabZ enzyme inhibition	
IC_50 _(μM)	9.7 ± 1.0
Inhibition type	Competitive
*K*_i _(μM)	1.9 ± 0.3

*H. pylori *stain inhibition (MIC in μg/ml)	

*H. pylori *SS1	5
*H. pylori *ATCC	10

### Kinetic analysis of Emodin/HpFabZ binding by SPR technology

**Figure 2:**
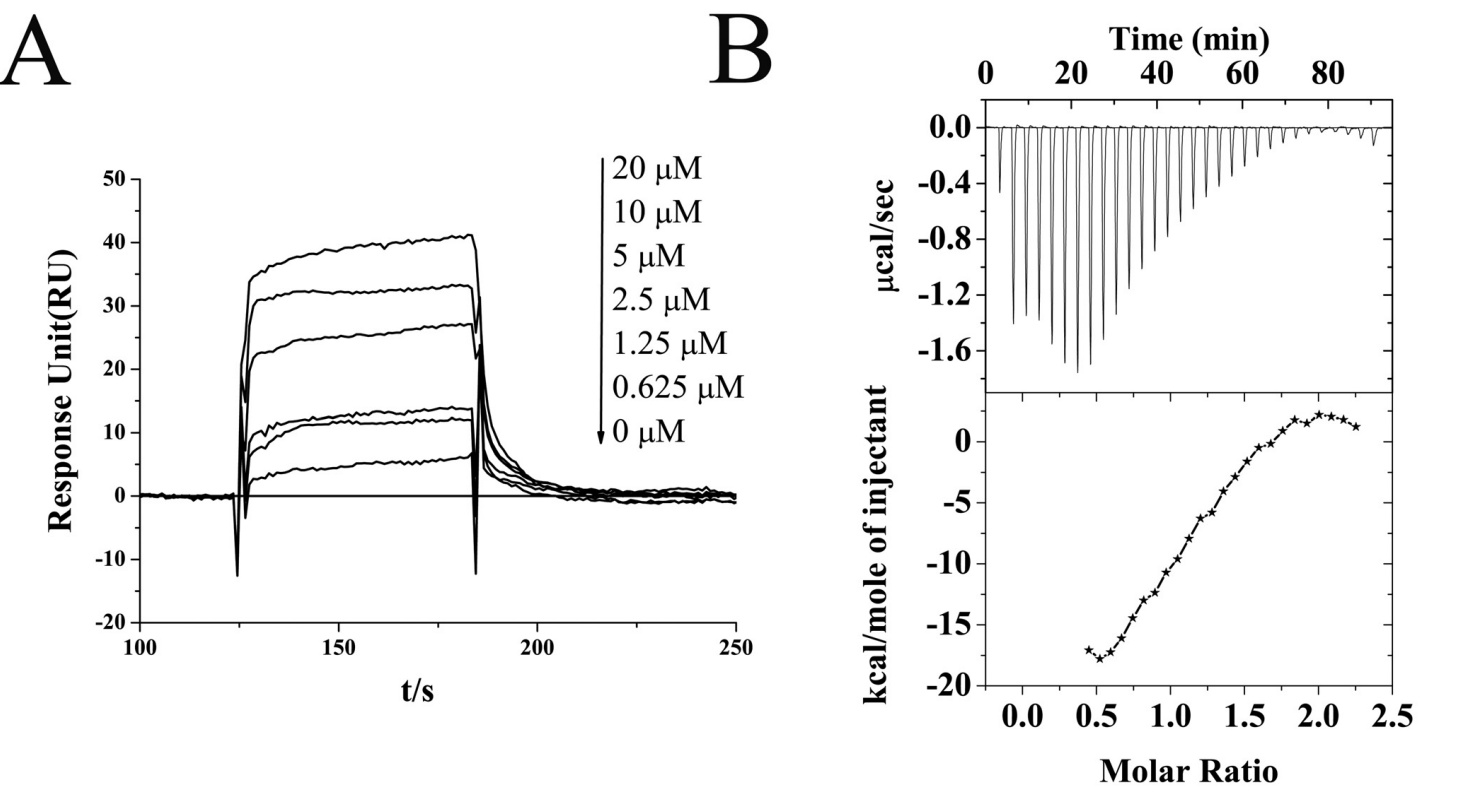
**(A) Sensorgrams of Emodin binding to HpFabZ measured by SPR technology based Biacore 3000 instrument**. Representative sensorgrams are obtained by injection of Emodin in varied concentrations of 0, 0.625, 1.25, 2.5, 5, 10, and 20 μM over HpFabZ that is immobilized on CM5 sensor chip. (B) ITC analysis of HpFabZ/Emodin interaction. Shown in Table 2 are the relevant thermodynamic parameters.

SPR technology based Biacore 3000 instrument was used to investigate the kinetic feature of Emodin binding to HpFabZ. In the assay, immobilization of HpFabZ on the Biacore biosensor chip resulted in a resonance signal of 6650 resonance units (RUs). The results in Fig. 2A indicated the dose-dependent biosensor RUs for Emodin, suggesting that this natural product could bind to HpFabZ *in vitro*.

**Table 2:** Kinetic and thermodynamic data of Emodin binding to HpFabZ

Kinetic Data*	
*R*_*max *_(RU)	42.3 ± 1.51
*k*_*a *_(per M per s)	4.21 × 10^4 ^± 0.273
*k*_*d *_(per s)	0.193 ± 0.0061
*K*_*D *_(μM)	4.59
Chi^2^	1.64

Thermodynamic Data**	

N	1.07 ± 0.035
*K*_*D*_' (μM)	0.45
ΔH (kcal/mol)	-17.77 ± 1.11
TΔS (kcal/mol)	-9.12

* *R*_*max*_, maximum analyte binding capacity; *K*_*a*_, association rate constant; *K*_*d*_, dissociation rate constant; *K*_*D*_, equilibrium dissociation constant determined by SPR; Chi^2^, statistical value in BIAevaluation; ** N, stoichiometry of Emodin-HpFabZ complex; *K*_*D*_', equilibrium dissociation constant determined by ITC.

The 1:1 Langmuir binding model was used to fit the kinetic parameters regarding the Emodin/HpFabZ binding process, in which the association rate constant (*k*_*a*_) and dissociation rate constant (*k*_*d*_) were fitted simultaneously by rate Equation 1,(1)

Where, *R *represents the response unit, *C *is the concentration of the Emodin, *R*_*max *_stands for the maximal response. The equilibrium dissociation constant (*K*_*D*_) was determined by Equation 2.(2)

The accuracy of the obtained results was evaluated by Chi^2^. The fitted kinetic parameters listed in Table 2 thus demonstrated a strong binding affinity of Emodin against HpFabZ by *K*_*D *_value of 4.59 μM, which is consistent with *K*_i _value.

### Thermodynamic analysis of Emodin/HpFabZ binding by isothermal titration calorimetry (ITC)

To inspect the kinetic and thermodynamic characters regarding the inhibition of Emodin against HpFabZ enzyme, ITC technology based assay was performed. Fig. 2B showed the raw data with subtraction of the blank titration. The ITC titration data in Table 2 has clearly established a 1:1 stoichiometry for HpFabZ-Emodin complex formation. Based on the obtained thermodynamic data (ΔH = -17.77 ± 1.11 kcal/mol, TΔS = -9.12 kcal/mol, ΔG = -8.65 kcal/mol), it was easily concluded that the enthalpy contributed favorably to the binding free energy in Emodin/HpFabZ interaction, indicating a significant enthalpy driven binding of Emodin to HpFabZ.

As shown in Table 2, Emodin exhibits a strong binding affinity against HpFabZ with *K*_*D*_' value of 0.45 μM fitted from ITC data.

It is noticed that the almost 10-fold difference between the KD values fitted from SPR and ITC based assays could be tentatively ascribed to the different states for HpFabZ. In SPR assay, HpFabZ was immobilized on CM5 chip, which might cause some conformation limitation for the enzyme. While in ITC assay, HpFabZ exists freely without any conformation restriction.

### Anti-*H. pylori *activity of Emodin

The inhibition activities of Emodin against *H. pylori *strains SS1 and ATCC 43504 were assayed according to the standard agar dilution method [31]. The MIC (minimum inhibitory concentration) value was defined as the lowest concentration of antimicrobial agent that completely inhibited visible bacterial growth. The results thus suggested that Emodin could inhibit the growth of *H. pylori *strains SS1 and ATCC 43504 with MIC values of 5 μg/ml and 10 μg/ml, respectively (Table 1).

### Crystal structure of HpFabZ-Emodin complex

The crystal structure of HpFabZ in complex with Emodin was determined to inspect the binding details of Emodin against HpFabZ at atomic level. HpFabZ-Emodin crystallization was performed using hanging-drop vapor-diffusion method and the crystallographic statistics are summarized in Table 3.

**Table 3:** Summary of diffraction data and structure refinement statistics

	HpFabZ-Emodin
Data collection	
Space group	P2_1_2_1_2_1_
Cell dimensions	
*a, b, c*(Å)	74.2036, 100.3975, 186.4314
α, β, γ (°)	90.00, 90.00, 90.00
Wavelength (Å)	1.5418
Resolution (Å)^1^	20-2.30 (2.42-2.30)
*R*_merge _(%)	12.4 (55.5)
*I*/σ*I*	18.8 (2.6)
Completeness (%)	99.6 (98.3)
Redundancy	8.9 (6.1)
Refinement	
Resolution (Å)	20-2.30 (2.42-2.30)
No. reflections	54135
*R*_work_/*R*_free_	0.199/0.233
No. atoms	
Protein	7274
Ligand/ion	69
Compound	40
Water	468
B-factors	
Protein	24.081
Ligand/ion	38.819
Water	29.006
Compound	42.133
R.m.s deviations	
Bond lengths (Å)	0.008
Bond angles (°)	1.4

^1^Numbers in parentheses represent statistics in highest resolution shell

In the complex structure, HpFabZ hexamer displayed a classical "trimer of dimers" organization similar to the native HpFabZ structure (PDB code 2GLL). Six monomers of the hexamer arranged a ring-like contact topology (A-B-F-E-C-D-A), and every two monomers (A/B, C/D and E/F) formed dimer each other through hydrophobic interactions. Two L-shaped substrate-binding tunnels with the entrance protected by a door residue Tyr100 were located in the interface of a dimer and ~20 Å away from each other. Tyr100 adopted two different conformations. The open conformation, in which the side chain of Tyr100 pointed towards Ile64' (the prime indicated the residue from the other subunit in the dimer), allowed the chains of substrates to enter the tunnel. While the closed conformation, in which the side chain of Tyr100 flopped ~120° around the C_α_-C_β _bond and pointed towards residue Pro112', blocked the entrance of the tunnel and stopped the substrate chain from reaching the catalytic site. The catalytic site in the tunnel was formed by two highly conserved residues, His58 and Glu72' that were located in the middle kink of the tunnel.

**Figure 3:**
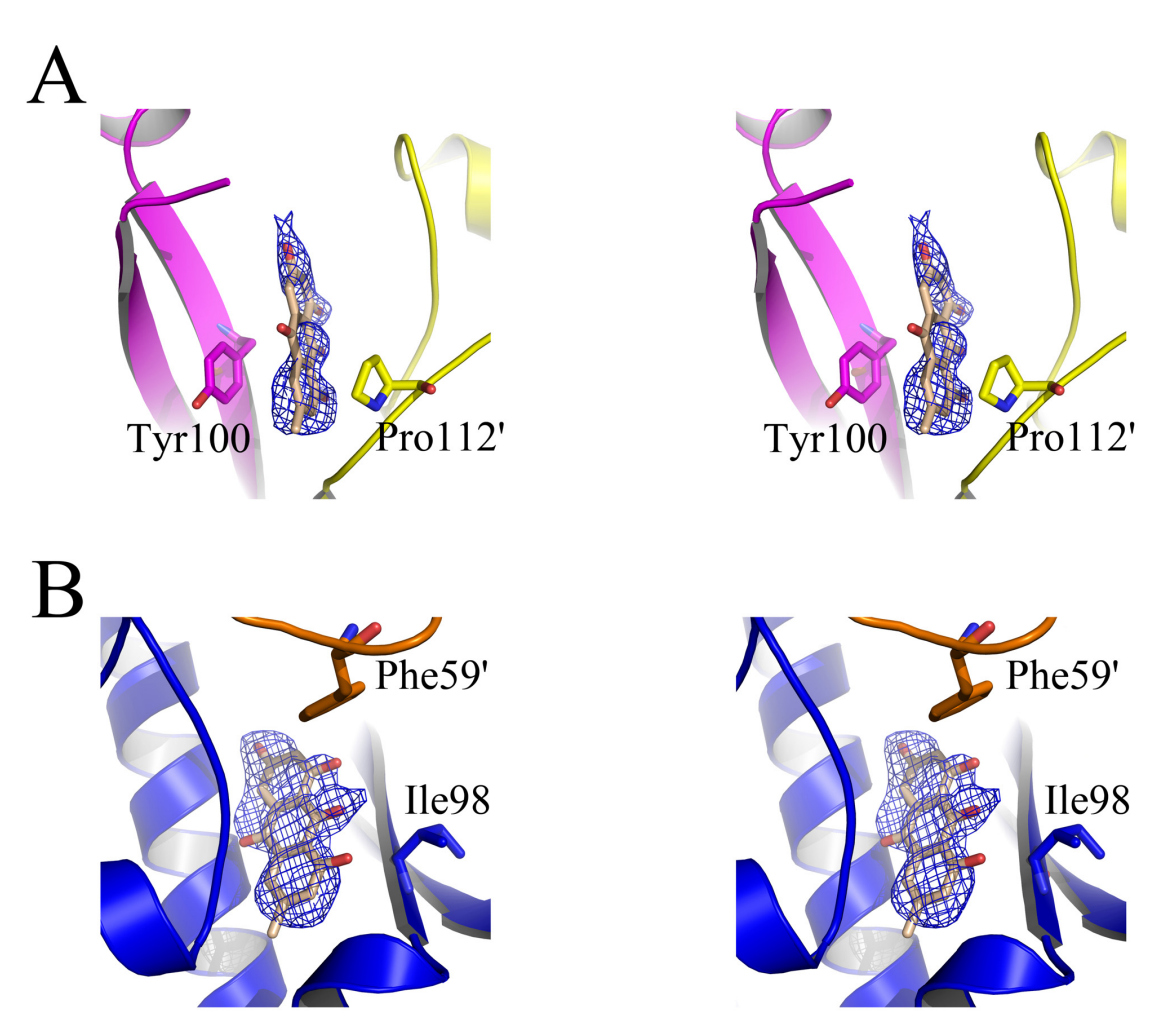
**Stereo view of the omit electron density map contoured at 1.0σ around Emodin**. Monomers A/B, C/D and Emodin are colored yellow/magenta, blue/orange and wheat, respectively. Residues interacted with Emodin are shown as sticks.

**Figure 4:**
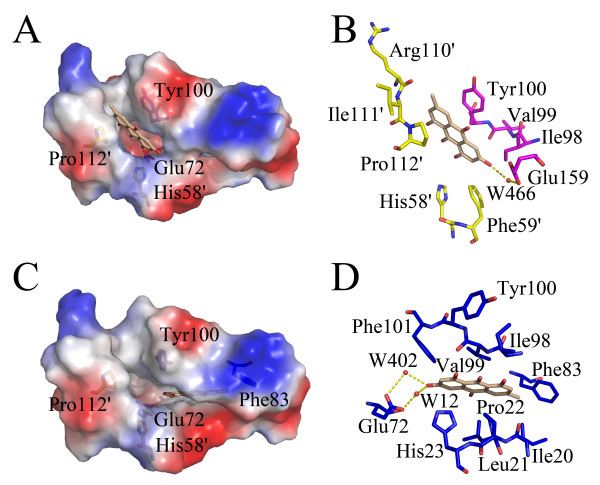
**Schematic diagram of Emodin binding models against HpFabZ**. The electrostatic surface of the active tunnel is rendered by a color ramp from red to blue. Emodin and surrounding critical residues are shown as sticks; water molecules that interact with Emodin are shown as red sphere. Hydrogen bonds are shown as yellow dashes. Emodin is colored wheat, and residues are colored in yellow, magenta, blue and orange for monomers A, B, C and D, respectively. The diagram was produced by the program Pymol. (A) Binding model A of Emodin around the entrance of tunnel B. Emodin binds to the entrance of tunnel B linearly through hydrophobic interactions, and is stacked between residues Tyr100 and Pro112'. (B) The interactions between Emodin and residues nearby (as well as some water molecules) in model A are indicated. Ring A of Emodin is stacked between Tyr100 and Pro112' forming a sandwich structure. 3'-methyl of ring A and C forms hydrophobic interactions with residues near the tunnel entrance. In addition, 6'-hydroxyl of ring C interacts with water molecule W466 through hydrogen bond. (C) Binding model B of Emodin near the catalytic site of tunnel C. Emodin extents to the bottom of the tunnel and is located in the hydrophobic pocket. (D) The interactions between Emodin and residues nearby (as well as some water molecules) in model B are indicated. The whole molecule of Emodin hydrophobic interacts with residues near by as well as hydrogen bonded interacts with waters W12 and W402 through its 6'-hydroxyl of ring C.

Emodin inhibited HpFabZ activity by either binding to Tyr100 or embedding into the middle of the tunnel C appropriately with favorable shape of complementary, thus preventing the substrate from accessing the active site. It bound to tunnels B and C of HpFabZ hexamer with two distinct interaction models, similar to the binding feature of HpFabZ-compound 1 complex (PDB code: 2GLP) [8] (Fig. 3). The two binding models were shown in Fig. 4. In one model (designated hereinafter as model A in Fig. 4A), Emodin bound to the entrance of tunnel B linearly (Tyr100 of the tunnel came from monomer B). Different from the open and close conformations, the phenol ring of door residue Tyr100 flopped ~120° to a third conformation and paralleled the pyrrolidine ring of Pro112'. Ring A of Emodin was then stacked between the phenol ring and pyrrolidine ring forming a sandwich structure, while 3'-methyl of ring A also interacted with residues Arg110 and Ile111 via hydrophobic interactions. Apart from the interactions between ring A and residues near the tunnel entrance, ring C of Emodin also formed Vander Waals interactions with residues Phe59' and Ile98, and was stabilized in the right place by the hydrogen bond interaction between 6'-hydroxyl of ring C and water molecule 466 which formed H-bond to Oε2 of Glu159 (Fig. 4B). In the other binding model (designated hereinafter as model B in Fig. 4C), Emodin entered into the middle of the tunnel C near the catalytic site, and located in the hydrophobic pocket consisting of residues Ile20, Leu21, Pro22, His23, Gly79, Phe83, Ile98, Val99 and Phe101. Ring A extended to the bottom of the tunnel and was stacked between residues Pro22 and Ile98, ring B interacted with residue Val99, while ring C bound to residues His23 and Phe101 through hydrophobic interactions. Additional hydrophobic interactions between 3'-methyl of ring A and residues Ile20 and Phe83, and hydrogen bond interactions between 6'-hydroxyl of ring C and water molecules of W12 and W402 which formed H-bonds to Oε1 and Oε2 of Glu72 respectively stabilized Emodin in the right place (Fig. 4D).

## Discussion

It is known that Emodin shows a wide range of pharmacological properties including anticancer, anti-inflammatory, antiproliferation, vasorelaxant and anti-*H. pylori *activities. However, to date no targeting information has been revealed regarding Emodin's anti-*H. pylori *activity. FabZ is an important enzyme responsible for elongation cycle of both saturated and unsaturated fatty acid biosynthesis in FAS II pathway that is essential for membrane formation in bacteria, and it has been recognized as an attractive target for antibacterial drug discovery [6]. Recently, the enzymatic characterization has been investigated for FabZ enzymes from several different strains including *Enterococcus faecalis *(EfFabZ) [32,33], *Pseudomonas aeruginosa *(PaFabZ) [34], *Plasmodium falciparum *(PfFabZ) [29,35], and *H. pylori *(HpFabZ) [7]. The crystal structural analyses have been determined for PaFabZ and PfFabZ [6,29,34], while some inhibitors against PaFabZ and HpFabZ were also discovered [8,29,30,36,37].

In the current work, the crystal structure of HpFabZ/Emodin complex was determined, and two different binding models (models A and B) were put forwarded. In the models, the hydrophobic interactions between Emodin and the nearby residues of HpFabZ contributed to the major interaction forces. In model A, the interaction between ring A of Emodin and residues Tyr100 and Pro112' in sandwich manner is the main hydrophobic interaction force, resulting in better electron density map around ring A, while ring C at the other end of Emodin had only weak interactions with residues nearby. In model B, the whole molecule of Emodin dove deeply into the active tunnel forming intense hydrophobic interactions with the residues nearby, thus the electron density map around Emodin was continuous, completive and much better than the map in model A (Fig. 3). Additionally, this interaction has also made the average B factor of Emodin in model B better than in model A (The average B factor of Emodin was 45.03 in model A, while 39.24 in model B).

**Figure 5:**
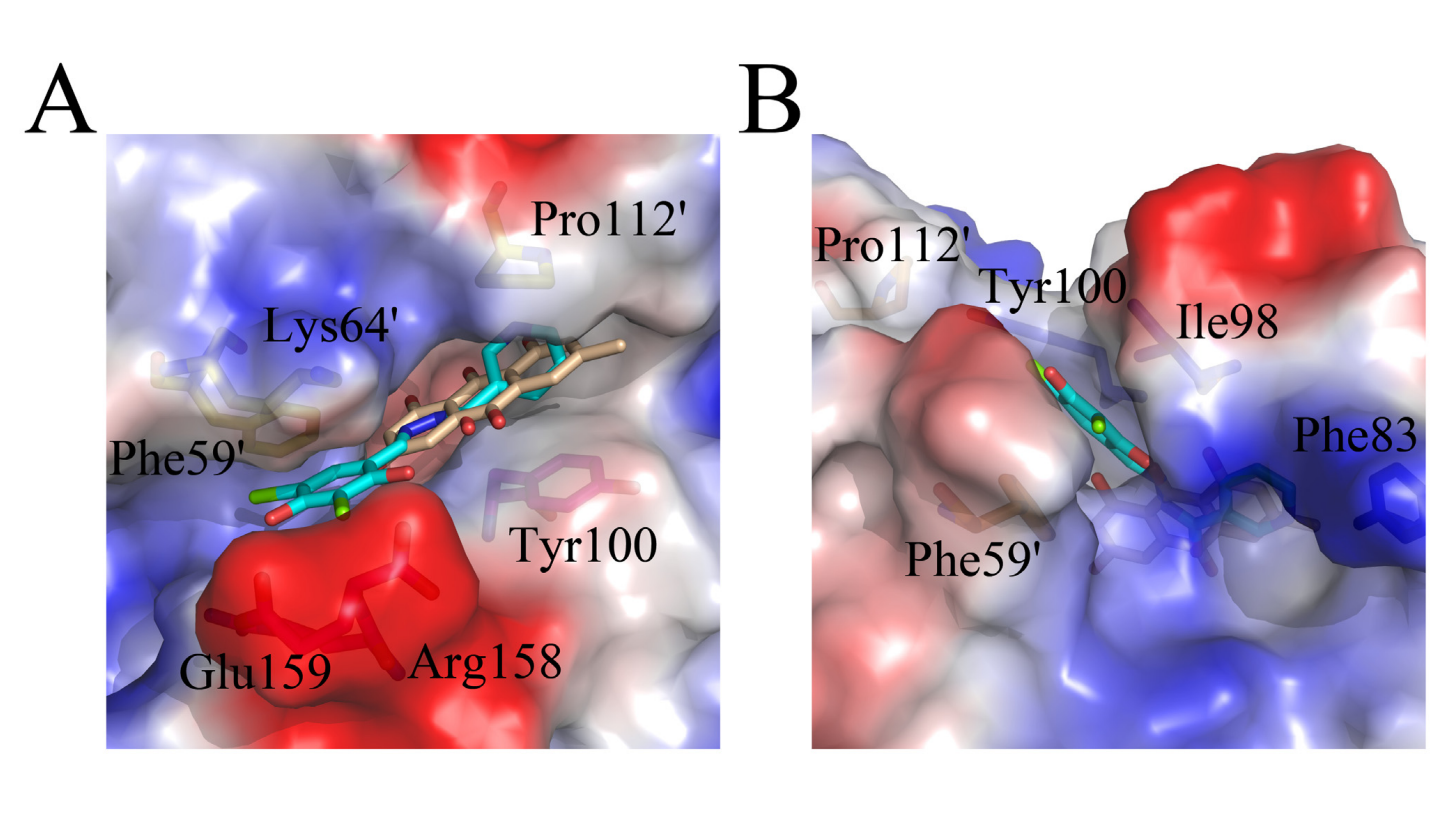
**The structure superposition diagram of Emodin and compound 1 in models A and B**. The electrostatic surface of the active tunnel is rendered by a color ramp from red to blue. Emodin, compound 1 and surrounding critical residues are shown as sticks and colored wheat, cyan, yellow (for monomer A), magenta (for monomer B), blue (for monomer C) and orange (for monomer D), respectively. Bromine on the compound 1 is colored green. (A) Emodin are located near the entrance of the active tunnel and stacked between Tyr100 and Pro112' in model A. The pyridine ring of compound 1 is also sandwiched as Emodin, while the 2,4-dihydroxy-3,5-dibromo phenyl ring at the other end of compound 1 stretches into another pocket formed by Arg158, Glu159, Phe59', Lys62' through hydrophobic interactions. (B) Emodin and compound 1 are located near the catalytic site of the active tunnel in model B. Emodin extents to the bottom of the tunnel and is located in the hydrophobic pocket. The pyridine ring of compound 1 adopts a similar conformation with Emodin. While the 2,4-dihydroxy-3,5-dibromo phenyl ring at the other end of compound 1 stretches out of the tunnel forming a sandwich conformation with residues Ile98 and Phe59' via π-π interactions.

In comparison with our recent published crystal structure of HpFabZ in complex with compound 1 (PDB code 2GLP) [8], there are some differences concerning their binding features due to the longer molecule of compound 1 than Emodin. In model A, the pyridine ring of compound 1 was sandwiched between residues Tyr100 and Pro112' linearly as ring A of Emodin, while the 2,4-dihydroxy-3,5-dibromo phenyl ring at the other end of compound 1 stretched into another pocket formed by Arg158, Glu159, Phe59', Lys62' through hydrophobic interactions, which can not be found in the binding model A of Emodin (Fig. 5A). In model B, compound 1 entered into the middle of the tunnel. Its pyridine ring accessed the end of the tunnel where the ring C of Emodin located in the model B, and stayed in the right place via hydrophobic interactions. However, the 2,4-dihydroxy-3,5-dibromo phenyl ring of compound 1 was too large to dive into the tunnel. Thus it had to adopt a crescent shaped conformation and stretched the 2,4-dihydroxy-3,5-dibromo phenyl ring out of the tunnel forming a sandwich conformation with residues Ile98 and Phe59' via π-π interactions. Based on these additional interactions, compound 1 should have a better inhibition activity against HpFabZ than Emodin. However, due to the poor solubility, compound 1 actually displayed higher B factor and lower IC_50 _value than Emodin.

The structural analysis indicated that the inhibitors specifically bound to tunnels B and C rather than the other four active tunnels of HpFabZ hexamer. As mentioned in our previous work [8], the crystal packing caused displacements of β3 and β6 strands in monomers B and C which made the hydrophobic active tunnel exposed to the bulk solvent. The hydrophobic surroundings then promoted the binding of the inhibitors.

As reported [38], ITC technology based analysis can provide valuable information regarding the partition between enthalpy and entropy thus for lead compound optimization reference. Usually, it is proposed that entropy-driven ligand, characterized by a huge and favorable entropic contribution is prone to drug resistance, while the enthalpy-driven one might be the preferred starting point for lead optimization. As far as the Emodin/HpFabZ interaction is concerned, the enthalpy contributed favorably to the binding free energy (Table 2), thereby implying that Emodin might be propitious to the further structure modification as a lead compound. Of note, ITC result has suggested that Emodin binds to HpFabZ by a relative molar ratio of 1:1 in solution (Fig. 2), which seems to be a little paradoxical to the Emodin binding state in Emodin/HpFabZ complex crystal structure, where Emodin specifically bound to tunnels B and C of HpFabZ hexamer by a 1:3 stoichiometric binding mode (Emodin/HpFabZ). We tentatively ascribe such a discrepancy to the complex crystal formation that is different from the solution state. In the complex crystal through Emodin soaking method, the displacements of β3 and β6 strands in monomers B and C might promote the binding of Emodin, while the active tunnels of the rest four monomers with no displacement in β3 strand were completely blocked by the surface, thus interfering with the Emodin entry into the active tunnel to form co-crystal. But in solution, six monomers were highly symmetric and the β3 strands might exhibit much more flexible conformation to allow Emodin to enter into the active tunnels of all the six monomers, resulting in a 1:1 stoichiometry for HpFabZ/Emodin complex formation.

In addition, we also confirmed that Emodin could inhibit the growth of *H. pylori *strains SS1 (MIC: 5 μg/ml) and ATCC 43504 (MIC: 10 μg/ml). We could thereby suppose that the inhibition against HpFabZ might be one of the key factors for its *H. plori *strain inhibition, although there are maybe other undiscovered acting targets for Emodin.

Recently, apart from Emodin, some other HpFabZ inhibitors have been discovered to inhibit the growth of *H. pylori*. For example, Juglone, a natural product, was reported to inhibit the growth of *H. pylori *strains SS1 with MIC value of 5 μg/ml [36]. Three flavonoids (Quercetin, Apigenin and (S)-Sakuranetin) inhibited *H. pylori *strains ATCC 43504 at MIC values of 100, 25, 25 μg/ml, respectively [37]. All these inhibitors shared the same competitive inhibition mechanism against HpFabZ and bound to the same residues of the binding site from HpFabZ.

## Conclusion

Summarily, Emodin was firstly discovered as a competitive inhibitor against HpFabZ. The kinetic and thermodynamic characterization of Emodin/HpFabZ interaction has been completely performed by SPR and ITC based assays. The analyzed HpFabZ/Emodin complex crystal structure has clearly suggested that the inhibition of Emodin against HpFabZ could be carried out either by its occupying the entrance of the tunnel or plugging the tunnel to prevent the substrate from accessing the active site. Our work is expected to shed light on the potential inhibitory mechanism of Emodin against HpFabZ, while Emodin has been suggested to be a potential lead compound for further anti-bacterial drug discovery.

## Abbreviations

Emodin: 3-methyl-1, 6, 8-trihydroxyanthraquinone; anti-*H. pylori*: anti-*Helicobacter pylori*; HpFabZ: β-hydroxyacyl-ACP dehydratase from *Helicobacter pylori*; SPR: surface plasmon resonance; ITC: isothermal titration calorimetry; Hp: *Helicobacter pylori*; FAS II: the type II fatty acid synthetic pathway; FabZ: β-hydroxyacyl-ACP; TCM: traditional Chinese medicine; VEGF: vascular endothelial growth factor; DMSO: Dimethyl sulfoxide; MR: molecular replacement; MIC: minimum inhibitory concentration; RUs: resonance units; *k*_*a*_: association rate constant; *k*_*d*_: dissociation rate constant; *K*_*D*_: equilibrium dissociation constant.

## Authors' contributions

This study was designed by JC, LZ YG and XS. The kinetic and thermodynamic assays were performed by JC. Emodin inhibition against HpFabZ and *H. pylori *activity were performed by LZ and YZ. HpFabZ-Emodin complex crystallization, data collection, Structure determination and refinement were performed by LZ and HZ. JD assisted in the crystal data collection experiment. XS, YG, JD, HJ supervised the project. JC, LZ and XS contributed to the manuscript writing. All authors read and approved the final manuscript.

## Supplementary Material

Additional file 1 **Supplemental Materials**. Supplemental Figure Legends. Click here for file

Additional file 2 **Supplemental Figure S1**. pH profile of HpFabZ enzyme activity. Click here for file

Additional file 3 S**upplemental Figure S2**. The effect of DMSO on HpFabZ enzyme activity. Click here for file
