# Supplementary material for: Emodin targets the β-hydroxyacyl-acyl carrier protein dehydratase from Helicobacter pylori: enzymatic inhibition assay with crystal structural and thermodynamic characterization
Source: BMC Microbiol. 2009 May 12;9:91. doi: 10.1186/1471-2180-9-91 (PMC2692856; doi:10.1186/1471-2180-9-91)
Supplement: Additional file 1 — Supplemental Materials. Supplemental Figure Legends. [file 1471-2180-9-91-S1.doc]

***Supplemental Materials***

**Emodin targets the β-hydroxyacyl-acyl carrier protein dehydratase from *Helicobacter pylori*: enzymatic inhibition assay with crystal structural and thermodynamic characterization**

Jing Chen^‡1^, Liang Zhang^‡1^, Yu Zhang^1^, Haitao Zhang^1^, Jiamu Du^2^, Jianping Ding^2^, Yuewei Guo^1^*, Hualiang Jiang^1^ and Xu Shen^1^*

^1^Drug Discovery and Design Center, State Key Laboratory of Drug Research, Shanghai Institute of Materia Medica, Chinese Academy of Sciences, Shanghai 201203; ^2^Institute of Biochemistry and Cell Biology, Chinese Academy of Sciences, Shanghai 200031, China.

^‡^These two authors contributed equally to this work.

*Corresponding authors.

Phone&Fax: +86-21-50806918 (X. Shen). E-mail: xshen@mail.shcnc.ac.cn (X. Shen) and [ywguo@mail.shcnc.ac.cn](mailto:ywguo@mail.shcnc.ac.cn) (Y. Guo)

**Figure Legends**

**Fig. S1 pH profile of HpFabZ enzyme activity.** The HpFabZ enzyme activities were measured in the Bis-tris buffer with different pH (pH4.93, pH6.18, pH8.08, pH8.93, pH10.04).

**Fig. S2 The effect of DMSO on HpFabZ enzyme activity.**
