# Supplementary figures and images for: Emodin targets the β-hydroxyacyl-acyl carrier protein dehydratase from Helicobacter pylori: enzymatic inhibition assay with crystal structural and thermodynamic characterization
Source: BMC Microbiol. 2009 May 12;9:91. doi: 10.1186/1471-2180-9-91 (PMC2692856; doi:10.1186/1471-2180-9-91)

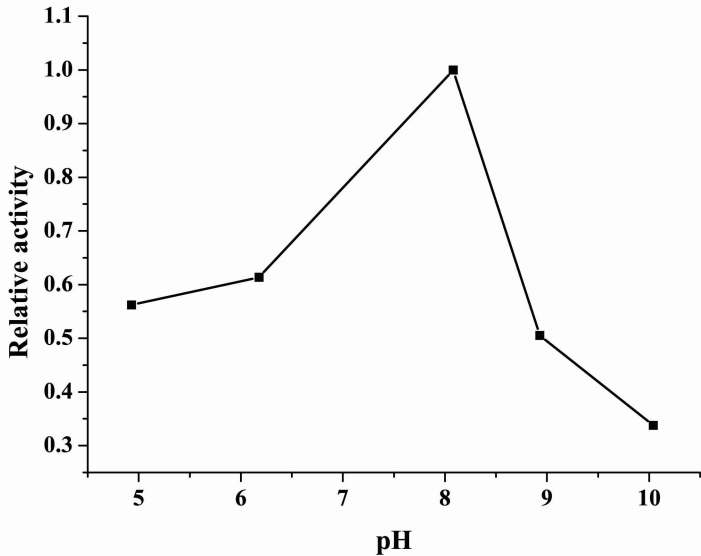

Supplement: Additional file 2 — Supplemental Figure S1. pH profile of HpFabZ enzyme activity. [file 1471-2180-9-91-S2.pdf]

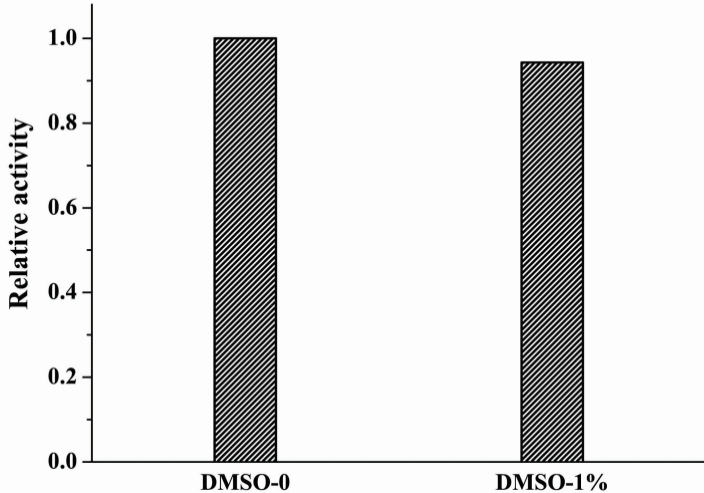

Supplement: Additional file 3 — Supplemental Figure S2. The effect of DMSO on HpFabZ enzyme activity. [file 1471-2180-9-91-S3.pdf]
